# Draft Genome Sequences of Six Strains Isolated from the Rhizosphere of Wheat Grown in Cadmium-Contaminated Soil

**DOI:** 10.1128/MRA.00676-20

**Published:** 2020-08-20

**Authors:** Vira Hovorukha, Ankita Bhattacharyya, Olga Iungin, Hanna Tashyreva, Victoria Romanovska, Olesia Havryliuk, Olena Bielikova, Claire Blackwell, Brian Burks, Cara Cothern, Jakia Elliott, Jonathan Hoover, Alexis Jones, Christian Leise, Linda Lowmiller, Ahmed Mohamed, Tiffany Mullen, Ethan Nettleton, Karshanda Polk, Benny Tran, Teresa Tran, Manuel Vega, Landon Ware, Emily Welch, Leandra Williams, Madison Woodard, Kaylin Young, Olga Mavrodi, Oleksandr Tashyrev, Dmitri Mavrodi

**Affiliations:** a Zabolotny Institute of Microbiology and Virology, National Academy of Sciences of Ukraine, Kyiv, Ukraine; b School of Biological, Environmental, and Earth Sciences, University of Southern Mississippi, Hattiesburg, Mississippi, USA; c Institute of Molecular Biology and Genetics, National Academy of Sciences of Ukraine, Kyiv, Ukraine; d South Mississippi Branch Experiment Station, Mississippi State University, Poplarville, Mississippi, USA; Indiana University, Bloomington

## Abstract

This study presents high-quality draft genome assemblies of six bacterial strains isolated from the roots of wheat grown in soil contaminated with cadmium. The results of this study will help to elucidate at the molecular level how heavy metals affect interactions between beneficial rhizobacteria and crop plants.

## ANNOUNCEMENT

Soil deterioration caused by intensive farming practices presents a serious challenge to agriculture in Ukraine and worldwide (1–3). Beneficial rhizobacteria play a crucial role in the remediation of contaminated soils and contribute to plants’ ability to resist soil pollution (4, 5). In this study, we characterized the genomes of six bacterial strains isolated from the rhizosphere of wheat grown in soil contaminated with cadmium, a heavy metal pollutant commonly associated with the extensive use of phosphate fertilizers (6).

Winter wheat (cv. Benefis) was grown for 3 weeks in cadmium-contaminated soil collected near Kyiv, Ukraine, and rhizobacteria were isolated by the dilution plating of root wash onto nutrient agar (Difco) supplemented with 75 μg ml^−1^ of CdCl_2_. Six isolates representing the dominant morphotypes, designated USM1 through USM6, were selected for genome sequencing and cultured in nutrient broth (Difco) prior to DNA extraction using a cetyltrimethylammonium bromide (CTAB) procedure (7). Sequencing libraries were prepared using a NEBNext Ultra II FS kit (New England Biolabs) and sequenced on an Illumina MiSeq instrument with v.3 chemistry, which generated between 1.7 and 3.2 million 150-bp paired-end reads per strain. The raw reads were filtered using FastQC (https://www.bioinformatics.babraham.ac.uk/projects/fastqc/), trimmed with TrimGalore v.0.6.1 (8), and assembled with Unicycler (9) or SPAdes v.3.12.0 (10) implemented in the Pathosystems Resource Integration Center (PATRIC) (11). The genome assemblies were assessed with QUAST v.5.0.2 (12) and annotated with the NCBI Prokaryotic Genome Annotation Pipeline (PGAP) (13). Genes involved in resistance to cadmium, other heavy metals, and biocides were identified by comparing the predicted proteomes to the BacMet database (14). Default parameters were maintained for all analyses, except in BacMet searches, which were performed using BLASTp v.2.10.0 (15) with a cutoff of e-06, identity of 40%, and coverage of 60%. The results of the genome annotation and other relevant information are listed in Table 1.

**TABLE 1:** Genome features of strains Brevundimonas vesicularis USM1, Pseudarthrobacter oxydans USM2, Pseudomonas lini USM3, Pseudomonas putida USM4, Cupriavidus gilardii USM5, and Cupriavidus taiwanensis USM6

Strain	No. of reads	Fold coverage (×)	Genome size (bp)	No. of contigs	*N*_50_ (bp)	G+C content (%)	Total no. of genes	No. of RNA genes	Total no. of CDSs^*a*^	No. of PECs^*b*^	No. of hypothetical proteins	No. of metal resistance genes^*c*^	GenBank accession no.	SRA accession no.
USM1	2,313,022	110	3,089,804	41	141,252	66.21	3,118	53	3,065	439	641	15	JABTYI000000000	SRR11881363
USM2	3,189,890	100	4,689,826	90	120,754	65.75	4,401	61	4,340	481	909	9	JABTYH000000000	SRR11881362
USM3	1,949,146	46	6,264,375	57	225,657	58.92	5,628	60	5,568	781	861	61	JABTYG000000000	SRR11881361
USM4	2,281,918	60	5,623,397	53	288,931	61.88	5,193	73	5,120	731	726	66	JABTYF000000000	SRR11881360
USM5	2,101,546	56	5,535,898	60	194,803	67.17	4,917	59	4,858	610	722	74	JABTYE000000000	SRR11881359
USM6	1,704,650	40	6,358,134	153	76,088	67.70	5,860	63	5,797	664	918	70	JABTYD000000000	SRR11881358

a CDSs, coding sequences with proteins.

b PECs, proteins with Enzyme Commission numbers.

c Predicted by BLASTp searches against the BacMet database.

**FIG 1:**
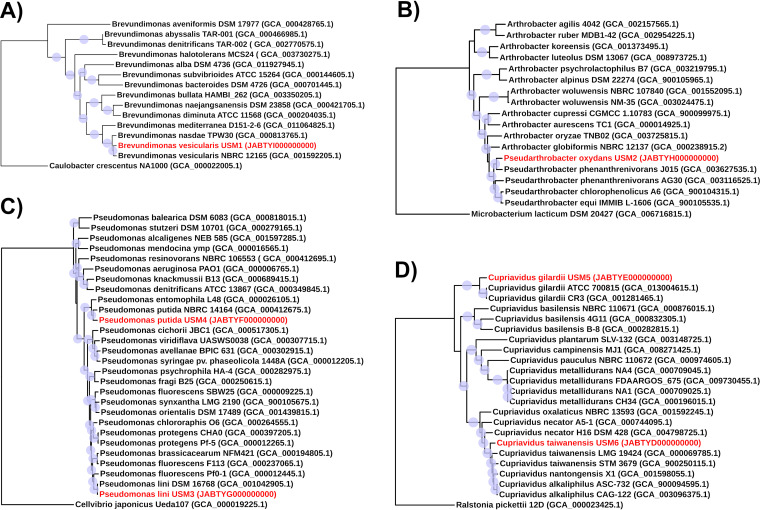
Whole-genome comparison of Brevundimonas vesicularis USM1 (A), Pseudarthrobacter oxydans USM2 (B), Pseudomonas lini USM3 and Pseudomonas putida USM4 (C), and Cupriavidus gilardii USM5 and Cupriavidus taiwanensis USM6 (D) to their closest relatives. The whole-genome phylogenies were inferred with the KBase SpeciesTreeBuilder (16) based on a selected subset of clusters of orthologous groups (COGs) and edited in iTOL (17). The genome sequences of Caulobacter crescentus NA1000, Microbacterium lacticum DSM 20427, Cellvibrio japonicus Ueda107, and Ralstonia pickettii 12D were used as outgroups. Blue circles on the tree nodes represent bootstrap values varying between 70% (smallest circle) and 100% (largest circle). Values in brackets indicate GenBank accession numbers.

The whole-genome phylogenetic analyses performed with the KBase SpeciesTreeBuilder (16) identified the studied strains as Brevundimonas vesicularis USM1, Pseudarthrobacter oxydans USM2, Pseudomonas lini USM3, Pseudomonas putida USM4, Cupriavidus gilardii USM5, and Cupriavidus taiwanensis USM6 (Fig. 1). Further genome analysis revealed the presence of multiple efflux systems (cation diffusion facilitator [CDF] family transporters, heavy metal resistance/nodulation/cell division [RND] family permeases, P-type ATPase family ion pumps) presumably involved in the resistance to cadmium and other heavy metals. The genomes also encoded a broad spectrum of pathways for the degradation of aromatic compounds, including the main ring-cleavage β-ketoadipate pathway with catechol (USM3, USM4, and USM6) and protocatechuate ortho-ring-cleavage (USM1, USM3, USM4, and USM6) branches, as well as the meta-ring-cleavage pathway catalyzed by type I catechol-2,3-dioxygenase (USM1). Other notable features that may contribute to the rhizosphere lifestyle of the studied strains include pathways for the synthesis of antimicrobial phenazine compounds (USM3), siderophores, auxins (USM2), insecticidal toxins (USM3), different protein secretion systems, plasmids, and prophages.

### Data availability.

The annotated genomes were deposited in NCBI’s GenBank under the accession numbers listed in Table 1. The table also lists the accession numbers of the raw reads deposited in the Sequence Read Archive (SRA). All genome annotation and SRA data are included in BioProject number PRJNA635988.
